# Induced and spontaneous uterine leiomyomas in animal models: a scoping review

**DOI:** 10.1590/acb414426

**Published:** 2026-08-03

**Authors:** Willane Bandeira de Sousa, João Nogueira, Kauã Manuel Costa Araújo, Lyvia Maria Rodrigues de Sousa Gomes, Maria do Socorro de Sousa Cartagenes, João Batista Santos Garcia

**Affiliations:** 1 Universidade Federal do Maranhão – Postgraduate Program in Health Sciences – São Luís (MA) – Brazil.; 2 Universidade Federal do Maranhão – Department of Medicine – São Luís (MA) – Brazil.; 3 Universidade Federal do Maranhão – Department of Physiological Sciences – São Luís (MA) – Brazil.

**Keywords:** Leiomyoma, Models, Animal, Scoping Review

## Abstract

**Purpose:**

To map the experimental models of induced and spontaneous uterine leiomyomas (UL) in animals, describing induction techniques, evaluation methods, advantages, limitations, and research gaps.

**Methods:**

We conducted a systematic review of studies published between 2004 and 2024 with in-vivo animal experiments reporting UL induction or spontaneous occurrence. Data were extracted on animal species, sample size, induction methods, confirmation techniques, and reported advantages or disadvantages.

**Results:**

A total of 56 studies were included. Rats and mice were the most frequently used species. Induction methods comprised hormonal administration, genetic predisposition, chemical exposure (monosodium glutamate), ischemic injury, external stimulation, cell transplantation, xenotransplantation, and spontaneous development. A critical finding that emerged was that 60.7% of the studies failed to report confirmation of UL induction. Among the 22 studies that reported confirmation, caliper measurement was the most frequently used technique. This widespread lack of methodological rigor significantly undermines the credibility and reproducibility of many published models.

**Conclusion:**

This scoping review highlights not only the heterogeneity of UL animal models and the lack of standardized induction and evaluation protocols, but also identifies a critical gap in induction validation reporting that compromises the foundation of preclinical research in this field.

## Introduction

Uterine leiomyomas (UL) are one of the most common gynecological diseases, affecting women of childbearing age, causing abnormal uterine bleeding, symptoms related to increased uterine volume, and fertility problems, significantly reducing the quality of life of patients and resulting in high costs for the health system^1^.

Although the ideal pharmacotherapy for treating UL has not been developed yet, nonsteroidal anti-inflammatory drugs, hormonal agents, and gonadotropin-releasing hormone (GnRH) agonists are currently the first line of treatment. In cases of symptomatic uterine fibroids that do not respond to medical treatment, invasive procedures such as uterine artery embolization, high-intensity focused ultrasound, myomectomy, or hysterectomy may be necessary, resulting in more costs, trauma, and risks to patients with UL, justifying the search for new clinical treatments^2^.

Preclinical research is essential in this context, starting with identifying the need for a new drug to treat a specific disease or improve an existing treatment. At this stage, scientists investigate compounds with therapeutic potential through laboratory studies, molecular screening, and computational modeling^3^. The promising compounds are tested in cultured cells and animal models to assess efficacy and safety^4^. Promising drugs undergo rigorous testing to assess their toxicity in different organs and systems. If positive results are obtained, the development of the pharmaceutical form (tablets, capsules, or injections) and appropriate dosage begin, stages that precede clinical trials^5^.

Despite the crucial role of animal models in preclinical research, current literature on UL models exhibits significant heterogeneity in induction techniques, confirmation protocols, and evaluation methods, with a conspicuous lack of standardization across studies. This fragmentation hinders the ability of researchers to compare findings, select appropriate models for specific research objectives, identify best practices, and critically assess methodological quality, ultimately compromising the translational value of published research. To address this knowledge gap, a comprehensive mapping of available experimental models, their induction strategies, and validation approaches is urgently needed to guide researchers in model selection and promote standardization within the field.

Based on these premises, this study aimed to investigate the induction or spontaneous growth, evaluation methods, and advantages and disadvantages of UL in animal models, as well as to contribute to future research on clinical treatments of the disease.

## Methods

### Study design and registration

This study was conducted as a scoping review, guided by the Preferred Reporting Items for Systematic Reviews and Meta-Analyses extension for Scoping Reviews (PRISMA-ScR).

To ensure transparency, reproducibility, and to avoid duplication of efforts, the protocol was prospectively registered in the International Prospective Register of Systematic Reviews (PROSPERO), under ID No. CRD42025587878 on January 8, 2025.

### Research question

Using the Population, Concept, Context (PCC) framework, the guiding questions were: what experimental animal models of UL have been described in the literature?; and what are their induction methods, evaluation techniques, advantages, and limitations?

- Population (P): *in-vivo* animal models;
- Concept (C): induced or spontaneous UL;
- Context (C): experimental studies published between 2004 and 2024.

### Eligibility criteria

The analysis included randomized and observational *in-vivo* studies reporting UL induction or spontaneous occurrence in animals published between 2004 and 2024. Reviews, meta-analyses, case reports, conference abstracts, commentaries, books, and grey literature were excluded. Duplicate records and studies without full-text access were removed.

### Sources and search strategy

The search was conducted between November 2024 and February 2025 in the PubMed, Embase, Scopus, and Scientific Electronic Library Online (SciELO) databases. The search terms included were: “induced,” “leiomyoma,” “myoma,” “uterine fibroids,” “smooth muscle tumors,” and “animal model,” combined with Boolean operators AND and OR (Table 1).

**Table 1:** Search strategy.

Database	Search Strategy	Studies (n)
PubMed	(("leiomyoma"[MeSH Terms] OR "leiomyoma"[All Fields] OR "leiomyomas"[All Fields] OR ("myoma"[MeSH Terms] OR "myoma"[All Fields] OR "myomas"[All Fields] OR "myoma s"[All Fields]) OR "uterine fibroids"[All Fields] OR "smooth muscle tumors"[All Fields]) AND ("animal*"[All Fields] OR "model*"[All Fields])) AND (2004/1/1:2024/12/31[pdat])	1,771
Embase	('leiomyoma' OR 'leiomyoma'/exp OR leiomyoma OR 'myoma' OR 'myoma'/exp OR myoma OR 'uterine fibroids' OR 'smooth muscle tumors') AND ('animal model'/exp OR 'animal model') AND [2004-2024]/py	581
Scopus	(leiomyoma OR myoma OR 'uterine fibroids' OR 'smooth muscle tumors') AND (animal* OR model*)	438
SciELO	(leiomyoma OR myoma OR "uterine fibroids" OR "smooth muscle tumors") AND (animal* OR model*)	12

SciELO: Scientific Electronic Library Online. Source: Elaborated by the authors.

### Data collection process

Initially, the titles were analyzed, and the selected studies were extracted from each platform in .cvs format. Duplicates were removed, titles and abstracts were read, and those not meeting the eligibility criteria were removed. Then, the eligible studies were thoroughly evaluated. Two examiners were involved in the search, selection, and complete analysis of the articles. The data were compared, and a third examiner was consulted in case of disagreement or divergence.

### Data registration

A database was created in table format using Microsoft Excel 2019. Data were extracted into a structured table including study design, animal species, sample size, induction method, confirmation technique, and reported advantages or disadvantages.

### Synthesis of results

Findings were summarized descriptively and organized into categories of induction methods (hormonal, genetic, chemical, ischemic, external stimulation, transplantation, xenotransplantation, and spontaneous occurrence), as well as the mechanism for evaluating the induction proof, among the studies that reported on it.

## Results

### Study selection

**Figure 1:**
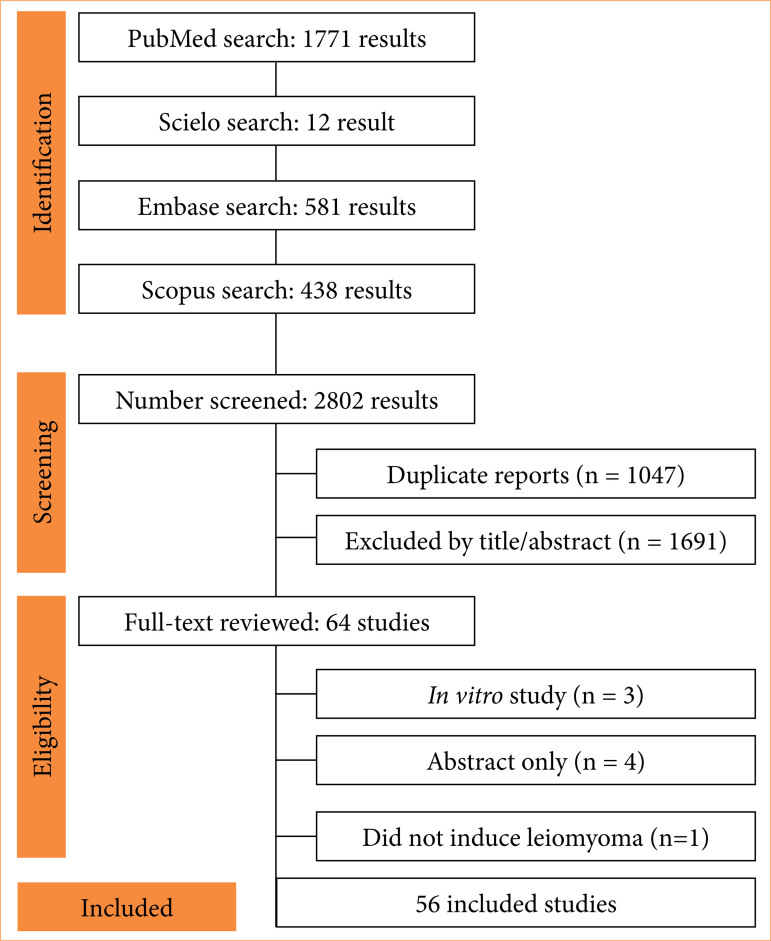
Flow diagram of evidence retrieval and selection according to Preferred Reporting Items for Systematic Reviews and Meta-Analyses extension for Scoping Reviews (PRISMA-ScR) guidelines.

Source: Elaborated by the authors.

After applying the inclusion criteria, it was found that, among the 2,802 articles in the search, 1,047 were duplicates, and 1,691 were excluded after reading the title and abstract. Furthermore, through careful analysis of the remaining 64 articles, 56 eligible studies were identified (Fig. 1).

### Synthesis of results

A total of 56 studies were included in this scoping review, comprising diverse animal models and methods of UL induction or spontaneous occurrence. Among those, 1,078 rats, 570 mice, 83 guinea pigs, 106 potbellied pigs, 313 hens, 196 chimpanzees, 217 rhesus macaques, and a single squirrel monkey were evaluated. Most studies (n = 37) used hormonal induction as a stand-alone method (n = 19) or combined with other techniques (n = 18), such as xenotransplantation or cellular implantation, followed by xenograft models (n = 10), transgenic or genetically predisposed animals (n = 2), monosodium glutamate (MSG) administration (n = 5), ischemic injury (n = 1), and external stimulation (n = 2), and seven studies investigated spontaneous UL development in non-human primates, pigs, and poultry. The majority (n = 34) did not report how experimental UL induction was confirmed. Among the studies that did report it (n = 22), the use of a digital caliper was the most frequently applied technique (n = 9) (Table 2).

**Table 2:** Characteristics of the included studies.

Authors	Year	Population	Randomization	Induction type	Proof of the induction method	Tumor characteristics
Elkafas et al.^6^	2020	10 Eker rats (Long Evans; Tsc2 Ek /+)	2 groups	Transgenic animals + Hormonal	Not specified	Accumulation of DNA damage (γH2AX)* and reduced expression of repair proteins (RAD50, MRE11, NBS1) in myometrial stem cells*
Yang et al.^7^	2023	10 Eker rats (Long Evans; Tsc2 Ek /+)	2 groups	Transgenic animals + Hormonal	Not specified	Fibrosis** and inflammatory infiltration of macrophages (CD68+) associated with hypomethylation of the Stat3 gene*
Yasong et al.^9^	2016	50 Sprague Dawley rats	5 groups	Hormonal	Not specified	Thickening of the smooth muscle layer with cellular disarray, polymorphic nuclei and large mitochondria**; overexpression of MMP-2 and reduction of TIMP-2*
Ju and Xiao^10^	2016	60 Wistar rats	6 groups	Hormonal	Not specified	Macroscopic thickening of the uterine horns, smooth muscle hyperplasia with cellular disarray and degeneration*; overexpression of ER, PR and Bcl-2 with reduction of Bax
Liu et al.^11^	2017	C57B6 mice	4 groups	Hormonal	Not specified	Significant thickening of the smooth muscle layer with cellular disarray and pleomorphic nuclei**; overexpression of Bcl-2 and reduction of Bax and Caspase-3*
Li et al.^12^	2019	Wistar rats	5 groups	Hormonal	Not specified	Uterine hyperplasia evidenced by a significant increase in the uterine coefficient and cervical diameter*; overexpression of Bcl-2, Akt, p-Akt and increased NOS activity, concomitant with a reduction in Bax and Caspase-3*
Li et al.^13^	2019	40 Sprague Dawley rats	4 groups	Hormonal	Not specified	Uterine hyperplasia evidenced by a significant increase in the uterine coefficient, transverse diameter and smooth muscle thickness*; overexpression of TGF-β3 and MMP-11 proteins, increased arachidonic acid and lysophosphatidylcholine associated with inflammation and abnormal angiogenesis*
Lin et al.^14^	2019	ICR (CD-1) mice	3 groups	Hormonal	Not specified	Smooth muscle hyperproliferation characterized by a significant increase in the thickness of the myometrial and endometrial layers, with cellular hypertrophy and irregular arrangement of muscle fibers*; overexpression of PCNA, ER-α, and PR*
Yu et al.^15^	2019	30 Sprague Dawley rats	6 groups	Hormonal	Not specified	Distorted and sparse nuclei with a "smudged" chromatin pattern and poorly defined contours, associated with a significant increase in extracellular matrix (ECM) thickness**; overexpression of genes linked to ECM formation (Lcn2, Aqp8) and proliferation, with dysregulation of MAPK, PPAR, Notch, and TGF-β pathways*
Lin et al.^16^	2020	70 specific pathogen-free rats	7 groups	Hormonal	Not specified	Hypertrophy and hyperplasia of smooth muscle cells**; intense endometrial proliferation and eosinophil infiltration, with the presence of hemorrhagic damage*; overexpression of PCNA, ER-α, ER-β, PR, Survivin, and Bcl-2, with a reduction in Bax and Caspase-3*
Olowofolahan et al.^17^	2020	24 Wistar rats	4 groups	Hormonal	Not specified	Disorganization of tissue architecture and a significant increase in fibroblast density*; overexpression of ER-α and β-catenin concomitant with loss of E-cadherin, suggesting activation of the Wnt pathway and epithelial-mesenchymal transition
Yamei et al.^18^	2021	140 Sprague Dawley rats	10 groups	Hormonal	Not specified	Thickening of the smooth muscle layer with cellular disarray and intense inflammatory infiltration**; overexpression of hormone receptors (ER-α, ER-β, PR)*
Yousefi et al.^19^	2021	84 Wistar rats	2 groups	Hormonal	Surgery + macroscopic analysis	Thickening of the uterine wall with disorganization of smooth muscle cells and extensive fibrosis**; increased expression and activity of MMP-2 and MMP-9, and oxidative stress (elevation of MDA and reduction of antioxidants SOD, CAT, GSH)*
Zhang et al.^20^	2021	27 Sprague Dawley rats	3 groups	Hormonal	Not specified	Focal hypertrophy and hyperplasia of smooth muscle cells with hyaline degeneration and vacuolization of epithelial cells*; increased tissue concentrations of E2, P, ER, and PR, in addition to increased plasma viscosity
Yu et al.^15^	2019	30 Sprague Dawley rats	6 groups	Hormonal	Not specified	Distorted and sparse nuclei with a "smudged" chromatin pattern and poorly defined contours, associated with a significant increase in extracellular matrix (ECM) thickness**; overexpression of genes linked to ECM formation (Lcn2, Aqp8) and proliferation, with dysregulation of MAPK, PPAR, Notch, and TGF-β pathways*
Lin et al.^16^	2020	70 specific pathogen-free rats	7 groups	Hormonal	Not specified	Hypertrophy and hyperplasia of smooth muscle cells**; intense endometrial proliferation and eosinophil infiltration, with the presence of hemorrhagic damage*; overexpression of PCNA, ER-α, ER-β, PR, Survivin, and Bcl-2, with a reduction in Bax and Caspase-3*
Olowofolahan et al.^17^	2020	24 Wistar rats	4 groups	Hormonal	Not specified	Disorganization of tissue architecture and a significant increase in fibroblast density*; overexpression of ER-α and β-catenin concomitant with loss of E-cadherin, suggesting activation of the Wnt pathway and epithelial-mesenchymal transition
Yamei et al.^18^	2021	140 Sprague Dawley rats	10 groups	Hormonal	Not specified	Thickening of the smooth muscle layer with cellular disarray and intense inflammatory infiltration**; overexpression of hormone receptors (ER-α, ER-β, PR)*
Yousefi et al.^19^	2021	84 Wistar rats	2 groups	Hormonal	Surgery + macroscopic analysis	Thickening of the uterine wall with disorganization of smooth muscle cells and extensive fibrosis**; increased expression and activity of MMP-2 and MMP-9, and oxidative stress (elevation of MDA and reduction of antioxidants SOD, CAT, GSH)*
Zhang et al.^20^	2021	27 Sprague Dawley rats	3 groups	Hormonal	Not specified	Focal hypertrophy and hyperplasia of smooth muscle cells with hyaline degeneration and vacuolization of epithelial cells*; increased tissue concentrations of E2, P, ER, and PR, in addition to increased plasma viscosity
Chen et al.^21^	2022	48 Sprague Dawley rats	6 groups	Hormonal	Surgical evaluation of three rats from each group	Thickening of smooth muscle with abnormally shaped/sized nuclei, disordered arrangement, and intense infiltration of inflammatory cells in the myometrium**; overexpression of Med12 protein and activation of the Wnt/beta-Catenin pathway, promoting cell proliferation
Qiu et al.^22^	2022	40 Sprague Dawley rats	5 groups	Hormonal	Not specified	Hypertrophy of smooth muscle cells with thickening of the muscle layer and extensive deposition of fibrous connective tissue between muscle bundles**; overexpression of IDO1, PI3KCA, and AKT1, inhibition of PTEN and apoptosis/autophagy markers (Caspase-9, LC-3), in addition to systemic immunosuppression (increased Tregs)*
Shi et al.^23^	2023	36 Sprague Dawley rats	6 groups	Hormonal	Histological evaluation of one rat from each group	Hyperplasia and disarray of smooth muscle cells with hypertrophic and dense nuclei, associated with inflammatory infiltration of neutrophils in the lamina propria*; overexpression of miR-21-5p and activation of the PI3K/AKT pathway (increased p-PI3K and p-AKT) concomitant with reduced PTEN*
Yuan et al.^24^	2023	108 Sprague Dawley rats	9 groups	Hormonal	Surgery + macroscopic analysis of two rats per group	Smooth muscle layer hyperplasia with cellular disarray, enlarged nuclei, connective tissue hyperplasia, and inflammatory infiltration**; elevated serum levels of E2, P, IL-6, and TNF-alpha and tissue overexpression of ER-alpha and PR*
Zeng et al.^25^	2023	6 Sprague Dawley rats	2 groups	Hormonal	Histopathological analysis of the model group	Thickening of the smooth muscle layer with focal and multiple hyperplasia, cellular hypertrophy, disordered arrangement, and connective tissue hyperplasia (fibrosis), associated with severe inflammatory infiltration**; overexpression of COX-2 and Bcl-2 concomitant with reduced Bax and elevated serum levels of E2 and P*
Gao et al.^26^	2024	48 Sprague Dawley rats	6 groups	Hormonal	Not specified	Smooth muscle hyperplasia (thickening) with disordered fiber arrangement, indistinct cell boundaries, and evident inflammatory infiltration**; overexpression of Bcl-2 concomitant with reduction of Bax and Caspase-3, in addition to systemic and tissue elevation of E2, P and ER
Yang et al.^27^	2023	196 Kunming mice	14 groups	Hormonal	Not specified	Hypertrophy and thickening of the smooth muscle layer with disordered arrangement of fibers, focal hyperplasia of the endometrium and infiltration of lymphocytes and eosinophils*; elevation of serum levels of E2, P, TNF-alpha and NOS activity, in addition to tissue overexpression of ER and PR*
Ezejiofor and Okoroafor^28^	2022	21 Wistar rats	3 groups	Monosodium glutamate	Not specified	Densely compacted fusiform fibrous tissue and multifocal tumor cells in the endometrium*; significant elevation of serum levels of E2 and P, in addition to markers of oxidative stress (increased MDA, reduced SOD, CAT, GSH) and renal insufficiency (increased urea and creatinine);
Imade et al.^29^	2024	25 Sprague Dawley rats	5 groups	Monosodium glutamate	Not specified	Thick bundles of spindle-shaped smooth muscle fibers arranged in a disordered and crisscrossed manner, invading the endometrial glands and stroma*; increased serum levels of total cholesterol and E2, indicating hormonal and lipid dysregulation associated with the tumor*
Kayode et al.^30^	2021	24 Wistar rats	4 groups	Monosodium glutamate	Not specified	Severe hyperplasia of endometrial spindle cells, disrupting the normal architecture of connective tissue*; elevated serum levels of E2, FSH, LH and total cholesterol*; high oxidative stress (elevated MDA and reduced SOD and CAT)*
Oyebode et al.^31^	2020	28 Wistar rats	4 groups	Monosodium glutamate	Not specified	Intense deposition of collagenous connective tissue in the myometrium and ovarian stroma, associated with a higher density of spindle fibroblasts**; elevated serum levels of E2, progesterone, total cholesterol, and triglycerides*
Mamoon et al.^32^	2021	33 Wistar rats	7 groups	Monosodium glutamate + Hormonal	Ultrasound with Dopplerfluxometry	Increased diameter and blood flow on Doppler ultrasound; proliferation of smooth muscle cells with excessive collagen deposition**; elevated serum levels of E2, progesterone (P4) and its receptors (ER, PGR), in addition to hemodynamic changes in the uterine arteries
Lin et al.^33^	2017	24 Wistar rats	3 groups	Ischemic injury	Not specified	Thickening of the myometrium and atrophy of the endometrium, with disarray of muscle fibers and significant accumulation of collagen**; overexpression of TGF-β, mast cells and activation of the Wnt/beta-Catenin pathway, promoting transdifferentiation of myofibroblasts*
Zhao et al.^35^	2018	Sprague Dawley rats	5 groups	Hormonal + external stimulation	Not specified	Focal hyperplasia of smooth muscle, hyaline and vacuolar degeneration, disordered arrangement and inflammatory infiltration**; elevated serum P and increased tissue levels of E2, P, ER, and PR*; NOTE: the group subjected only to stress (without hormones) did not develop tumor nodules, only functional alterations
Feng et al.^36^	2021	72 Sprague Dawley rats	9 groups	Hormonal + external stimulation	Not specified	Disarray of smooth muscle cells, indistinct cell boundaries and focal degeneration/necrosis**; overexpression of fibroblast activating protein (FAP) and TGF-beta, promoting excessive deposition of collagen I and activation of the AKT/ERK proliferation pathway
Abdelaziz et al.^37^	2016	18 naked mice	3 groups	ELT-3 + Hormonal injection	Physical examination + Digital caliper	Increased cell proliferation*; overexpression of extracellular matrix genes (TGF-beta) and angiogenesis (VEGF, IGF-1), in addition to Bcl-2 and PCNA*
Bar-Joseph et al.^38^	2020	14 specific pathogen-free rats	2 groups	ELT-3 + Hormonal injection	Digital caliper	Evident vascularization in the capsule and dependence on estrogen and VEGF for growth*; expression of estrogen receptors (alpha and beta), PEDF receptors (PNPLA2 and laminin) and VEGF production by tumor cells*
Chen et al.^39^	2019	10 nude mice (Foxn1nude)	2 groups	ELT3 injection	Digital caliper	High cell proliferation (PCNA overexpression) and intense extracellular matrix deposition (high levels of fibronectin and alpha-SMA), associated with an anti-apoptotic profile (reduced Bax/Bcl-2 ratio)
Halder et al.^40^	2014	24 athymic nude mice	3 groups	ELT-3 + Hormonal injection	Physical examination	High cellularity and intense extracellular matrix deposition (fibronectin and type i collagen overexpression)*; high cell proliferation (elevated PCNA, Mcm2 and Cyclin D1) and activation of critical signaling pathways, including TGF-beta, Wnt/beta-Catenin*; high expression of hormone receptors (ERα, PR-A, PR-B)*
Lin and Wu^41^	2022	14 BALB/C mice	2 groups	ELT3 injection	Digital caliper	High cell proliferation rate (elevated PCNA)*; constitutive activation of the AKT/mTORC1 survival and proliferation pathway and resistance to apoptosis (low levels of cleaved Caspase-3)*
Lin et al.^42^	2020	Not specified	2 groups	ELT3 injection	Not specified	High rate of cell proliferation (PCNA overexpression) and constitutive activation of the Shh/Gli1 signaling pathway (elevated levels of Sonic Hedgehog and Glioma-associated oncogene homolog 1 proteins)*
Rhee et al.^44^	2007	6 rabbits	1 group	VX2 injection	Magnetic resonance imaging + Ultrasound with Doppler flowmetry	Intense hypervascularization confirmed by angiography and enhancement on MRI; extensive central necrosis surrounded by viable tumor cells in the periphery**; NOTE: this is a cancer model used as a hemodynamic and not biological substitute for leiomyoma
Borahay et al.^47^	2015	20 immunodeficient mice NOG (NOD/Shi-scid/IL-2Rγnull)	2 groups	Xenograft + Hormonal	Digital caliper + Ultrasound	High rate of cell proliferation (high expression of the Ki67 marker)
Corachán et al.^48^	2020	48 NOD-SCID mice	6 groups	Xenograft + Hormonal	Digital caliper + PET/CT	Dense arrangements of smooth muscle fibers surrounded by an abundant collagenous extracellular matrix (ECM)**; rlevated expression of E2 and P receptors, in addition to active cell proliferation markers (Ki67 and PCNA) and high deposition of fibrotic proteins, specifically collagen I, fibronectin and PAI-1, regulated by the TGF-beta signaling pathway
Huang et al.^49^	2016	83 SCID mice	7 groups >> 3	Xenograft + Hormonal	Not specified	Tumor nodules adherent to the peritoneum with visible neovascularization; bundles of smooth muscle cells, significant increase in microvascular density and high VEGF expression**; upregulation of E2 and P receptors, high proliferative index evidenced by Ki67 nuclear staining*
Lee et al.^50^	2014	10 NOG mice (NOD+Shi / scid+IL-2Rγnull)	2 groups	Xenograft + Hormonal	Digital caliper + Physical examination	Bundles of spindle-shaped smooth muscle cells and organized collagenous extracellular matrix*; stable expression of proliferative markers (Ki67 and PCNA) and regulation of cell cycle (Cyclin D1) and anti-apoptotic (Bcl-2) proteins*
Li et al.^51^	2022	24 NOD-SCID mice	4 groups	Xenograft + Bisphenol A	Digital caliper	Morphology of solid nodules with proliferation dependent on activation of the XBP1/ITGA2 molecular axis*; upregulation of XBP1 and ITGA2, accompanied by activation of the PI3K/AKT signaling pathway (increased p-PI3K and p-AKT) and acceleration of the cell cycle*
Nair et al.^52^	2019	17 immunodeficient NOD-SCID mice	3 groups	Xenograft + Hormonal	Not specified	Bundles of spindle-shaped smooth muscle cells and significant collagen deposition in the extracellular matrix**; strong basal expression of proliferation markers (PCNA and Ki67) and maintenance of functional hormone receptors (ER and PR)*
Sousa et al.^53^	2015	6 Wistar rats	2 groups	Xenograft + Hormonal + immunosuppression	Surgery + macroscopic analysis	High graft survival rate, evidenced by the absence of necrosis and presence of active neovascularization; maintenance of the fibrotic component of the extracellular matrix, with 66.7٪ of peritoneal cases presenting grade III fibrosis and predominantly mild lymphocytic inflammatory infiltrate, without signs of cellular atypia or mitoses**
Suzuki et al.^54^	2018	39 BALB/C nu/nu mice	12 groups	Xenograft + Hormonal	Digital caliper	Fascicular architecture of smooth muscle cells and collagen-rich extracellular matrix, with evidence of peripheral growth mediated by synthetic/dedifferentiated cells*; overexpression of ER-alpha, ER-beta and PR receptors, activation of proliferation pathways (Ki67 and IGF2)*
Zakaria et al.^55^	2020	24 naked mice	4 groups >> 5	Xenograft	Not specified	Dense cellular organization with pleomorphic and hyperchromatic nuclei, presenting a disorganized fascicular arrangement typical of smooth muscle tumors with a high proliferative index*; induction of cell survival pathways, with a low rate of spontaneous apoptosis and maintenance of mitochondrial membrane integrity
Koohestani et al.^56^	2016	43 NOD-scid IL2Rynull mice	6 groups	Xenograft + Hormonal	Surgery on two rats per group	Dense bundles of smooth muscle cells and significant deposition of extracellular matrix**; elevated basal cell proliferation index (Ki67 positive), functional expression of ER and PR, marked presence of fibrotic proteins (collagen I and III) and expression of pro-fibrotic factors (TGF-beta, LOX3 and dermatopontin)*
Qiang et al.^57^	2014	NOD-scid IL2Rynull mice	Not specified	Xenograft + Hormonal	Not specified	Excessive accumulation of rigid ECM, composed predominantly of dense collagen I and III fibers, preserving the morphology of smooth muscle cell bundles**; consistent down-regulation of miR-29b, resulting in overexpression of pro-fibrotic target genes, including COL1A1 and COL3A1, in addition to maintaining stable expression of functional hormone receptors (ER and PR)*
Fritsch et al.^58^	2015	SCID mice	36 groups	Xenograft + Hormonal	Not specified	Intertwined bundles of fusiform smooth muscle cells and abundant deposition of disorganized collagenous ECM**; persistent expression of ER and PR, specific structural markers of smooth muscle (desmin and smooth muscle alpha-actin)*; high rates of active cell proliferation, evidenced by BrdU incorporation and Ki67 nuclear labeling*
Laik-Schandelmaier et al.^59^	2017	83 Guinea pigs	1 group	Spontaneous	Surgery on 64 animals	Bundles and whorls of spindle-shaped smooth muscle cells, endometrial adenomas, and malignant mixed Müllerian tumors*; molecular pathogenesis is strongly associated with a state of persistent hyperestrogenism, suggested by the massive coexistence of cysts in the rete ovarii and cystic follicles, which promote a continuous proliferative environment in the myometrium and endometrium
Mozzachio et al.^60^	2004	106 potbellied pigs	1 group	Spontaneous	Not specified	Firm, whitish, and often multifocal masses, with a fasciculated or spiral arrangement on the cut surface, ranging from microscopic nodules (2 mm) to voluminous masses (45 kg) located predominantly in the uterine horns*; intertwined bundles of well-differentiated leiomyocytes amidst an ECM with variable collagen deposition, which may exhibit hyaline degeneration and cystic cavitations*; strong cytoplasmic immunoreactivity for smooth muscle actin and low mitotic rates
Berry et al.^61^	2006	50 hens	1 group	Spontaneous	Not specified	Regions of active growth with prominent staining in connective tissue nuclei and greater proliferative intensity at the interface between normal tissue and tumor*; significant overexpression of ER and PR, in addition to a marked increase in PCNA and Bcl-2
Machado et al.^62^	2012	263 hens	1 group	Spontaneous	Not specified	Intertwined fascicles of spindle cells (arrangement in "thread ball" pattern) with abundant eosinophilic cytoplasm, elongated nuclei and absence of significant mitotic figures, showing hyaline degeneration and intense collagen deposition in the extracellular matrix*; smooth muscle phenotype (SMA and Desmin positive) accompanied by upregulation of ER-alpha and PR, increased BCL2 and elevated mRNA levels of profibrotic factors such as TGFB3 and type I collagen*
Silva et al.^63^	2006	1 chimpanzee	1 group	Spontaneous	Not specified	Whitish, firm and coalescent nodules that merge with the original myometrium*; well-differentiated neoplastic leiomyocyte bundles arranged in various directions, with abundant eosinophilic cytoplasm and elongated nuclei with dense chromatin, surrounded by moderately abundant and well-vascularized connective tissue*; strong and diffuse nuclear staining for ER and PR, accompanied by intense cytoplasmic reactivity for smooth muscle alpha-actin and low proliferative activity (few cells positive for MIB-1/Ki67)
Videan et al.^64^	2011	195 chimpanzees	1 group	Spontaneous	Physical examination + Transabdominal or transrectal ultrasound	Nodular masses detectable by ultrasound, laparoscopy or necropsy, frequently associated with clinical signs of excessive bleeding and anemia
Simmons and Mattison^65^	2011	217 monkeys rhesus	2 groups	Spontaneous	Not specified	Solid tumor masses in the myometrium*; intertwined bundles of spindle-shaped cells and occasional morphological variants, such as epithelioid leiomyoma or the presence of intratumoral neurilemmomas
Long et al.^66^	2010	1 Squirrel Monkey	1 group	Spontaneous	Physical examination + X-ray + Ultrasound + Exploratory laparotomy	Intertwined bundles of smooth muscle cells with sparse fibrous stroma, exhibiting elongated cells with poorly defined borders, "cigar-shaped" nuclei with single, small nucleoli, and an extremely low mitotic rate

* At the end of the study;

** findings confirming induction;

ER: estrogen receptor; PR: progesterone receptor; Bcl-2: B-cell lymphoma 2; Bax: Bcl-2-Associated X protein; PCNA: proliferating cell nuclear antigen; E2: estradiol; P: progesterone; Tregs: regulatory T cells; NOS: nitric oxide synthase; TGF: transforming growth factor; MMP: matrix metalloproteinase; TIMP: tissue inhibitor of metalloproteinases; ICR: Institute of Cancer Research; MDA: malondialdehyde; SOD: superoxide dismutase; CAT: catalase; GSH: glutathione; IL: interleukin; TNF: tumor necrosis factor; COX-2: cyclooxygenase-2; FSH: follicle-stimulating hormone; LH: luteinizing hormone; PGR: progesterone receptor; VEGF: vascular endothelial growth factor; IGF: insulin-like growth factor; PEDF: pigment epithelium-derived factor; MRI: magnetic resonance imaging; PET/CT: positron emission tomography with computed tomography; SCID: severe combined immunodeficiency.

Source: Elaborated by the authors.

Induced rodent models, particularly those using hormonal administration or MSG, stand out for their accessibility, low cost, and ease of handling, making them suitable for initial pharmacological screenings and basic mechanistic studies, despite limitations imposed by the lack of standardized protocols and the occurrence of adverse systemic effects that hinder direct extrapolation to human clinical practice. In contrast, specific models such as ischemic injury and VX2 tumor in rabbits serve specific investigative niches, respectively focused on the pathogenesis of fibrosis and the development of interventional radiology techniques (Table 3).

In terms of translational potential, xenotransplantation models and spontaneously occurring models (especially in non-human primates and swine) are superior, as they replicate the tissue architecture and hormonal responses observed in women. Xenotransplantation is consolidating as a robust tool for preclinical efficacy testing of new drugs directly in human tissue, although it requires complex infrastructure to maintain immunodeficient animals. Spontaneous models, while ideal for understanding the natural history of the disease due to high anatomical and genetic similarity, face significant ethical barriers and difficulties in access and standardization, which restrict their large-scale application (Table 3).

**Table 3:** Characteristics of the animal models.

Induction type	Species	Advantages	Limitations	Translational potential	Indication
Hormonal (estrogen +/- progestogen)	Rats (Wistar, Sprague Dawley) and Mice (Kunming, ICR, C57B6)	Simplicity, low cost, and no need for invasive surgical procedures. Allows precise control of doses and exposure time.	Prolonged use of high doses generates undesirable systemic effects (hepatic/renal). Lack of standardization of doses/routes and individual variability. Does not reflect the complexity of human genetics.	Low to moderate. Useful for initial screening, but too simplistic to mimic the full human disease.	Basic pharmacological investigations and mechanistic studies of hormonal response.
Transgenic + hormonal	Eker rats (Long Evans; Tsc2 Ek/+)	It mimics the interaction between a genetic (Tsc2 mutation) and an endocrine factor, closely resembling human etiopathogenesis.	It requires specific genetically modified cell lines. It also develops renal carcinomas (model specificity).	High. Relevant model for understanding genetic predisposition.	Studies of molecular mechanisms and gene-environment/hormone interaction.
Monosodium glutamate (MSG)	Rats (Wistar, Sprague Dawley)	Low cost, high availability, and oral administration (less invasive/stressful). Relatively short induction time (2–8 weeks).	The mechanism of action is not fully understood, making direct correlation with humans difficult. It requires intensive daily handling.	Low. The induction mechanism differs significantly from known human pathophysiology.	Laboratories with limited resources; basic studies of uterine hyperplasia.
Ischemic injury	Rats	It allows the study of the effects of hypoxia, fibrosis, and the vascular microenvironment on tumorigenesis.	Highly invasive and painful, compromising animal welfare. Ischemia affects other organs, generating confounding factors.	Specific. Relevant only to the post-injury fibrosis component.	Study of tissue fibrosis and myofibroblast differentiation.
External stimulus (stress)	Rats (Sprague Dawley)	It enhances hormonal induction by mimicking environmental/emotional factors (based on traditional Chinese medicine).	The methods resemble animal abuse (tail suspension, thermal shock, excessive noise).	Questionable. Based on premises of energy stagnation that are difficult to translate into Western medicine.	Studies on the impact of chronic stress and environmental factors on tumor growth.
Cell transplantation (ELT-3)	Nude/athymic (immunodeficient) mice	Fast, efficient, and less invasive than complex surgeries (injection only). Flexible (with or without hormone supplementation).	Dependence on the ELT-3 cell line (derived from mice) limits direct translation to humans. Cell culture logistics.	Average. Good for effectiveness, but it uses rodent-in-rodent cells.	In-vivo drug efficacy screening and cell proliferation studies.
VX2 tumor transplant	Rabbits	Larger animals, allowing the use of human medical devices (catheters). Does not require immunosuppression (healthy animal).	The VX2 tumor is a carcinoma (not a true leiomyoma), used only as a surrogate model for a vascularized mass.	High (for intervention). Excellent for testing surgical and interventional radiology techniques.	Studies of locoregional therapies (uterine artery embolization, ablation) and imaging.
Xenotransplantation (human tissue/cells)	SCID/NOG/Nude mice or immunosuppressed rats	It uses human tissue, reproducing real biological characteristics and therapeutic responses. It has significant translational relevance for drug testing.	High cost (immunodeficient animals), and complex, invasive surgical technique. Requires constant exogenous hormone supplementation.	Very high. This is the current gold standard for testing drugs intended for human use.	Preclinical efficacy trials of new drugs and personalized medicine.
Spontaneous	Non-human primates (chimpanzees, rhesus), pigs (potbellied) and chickens	Natural pathogenesis, high anatomical, histological and hormonal similarity to humans (especially primates and pigs).	Scarcity of detailed studies and difficulty in accessing animals (primates). Slow and unpredictable tumor development, hindering standardization.	Excellent. The most accurate model of the biology of human disease.	Studies of the natural history of the disease, its complex pathophysiology, and long-term therapeutic strategies.

ICR: Institute of Cancer Research; SCID: severe combined immunodeficiency. Source: Elaborated by the authors.

## Discussion

This scoping review mapped the available evidence on induced and spontaneous UL in animal models. The included studies revealed a wide diversity of species, induction methods, and evaluation techniques, reflecting the complexity of reproducing UL pathophysiology in experimental settings.

### Hormonal in transgenic animals

Elkafas et al.^6^ and Yang et al.^7^ used Eker rats (Long Evans; Tsc2 Ek/+), transgenic rats lineage genetically predisposed to develop UL and renal carcinomas due to mutations in the Tuberous Sclerosis 2 gene, to establish the model. To accelerate the development of the UL, 10 µg of diethylstilbestrol, a synthetic hormone derived from estrogen, was administered daily subcutaneously on days 10, 11, and 12 after birth, since this is a critical phase for the development of UL. The rats were euthanized after five months of life for evaluation.

Genetic predisposition combined with hormone administration mimics the endocrine and genetic factors associated with the development of UL in humans, allowing the investigation of specific molecular mechanisms, such as the interaction between genetic mutations and hormonal stimuli, offering a highly relevant model for translational studies^6,7^.

### Hormonal alone

Preclinical and clinical reports indicate that estradiol (E2) and progesterone (P) levels may serve as a key to the development and growth of UL^8^. The hormone-induced model alone was reported by 19 studies^9–27^.

Yasong et al.^9^ initially applied estrogen (0.5 mg/kg) intraperitoneally daily for four weeks in Wistar rats and then added progesterone at the same dose for five days, highlighting a progressive combined regimen.

One interesting fact about this model is the use of different dosages, routes of administration, and duration of estrogen and progesterone use. Ju and Xiao^10^ and Li et al.^12^ administered, in rats, diethylstilbestrol intramuscularly (IM) at a concentration of 2 mg/mL, with a dose of 0.02 mL daily for 30 days, while Yousefi et al.^19^ administered estradiol benzoate (0.2 mg/kg) IM, twice a week, for eight weeks. Olowofolahan et al.^17^ chose to administer estradiol valerate (3 mg/kg) by gavage daily for 12 weeks. In Kunming mice, Yang et al.^27^ divided the animals into three model groups and used estradiol benzoate (EB) in increasing doses of 0.3 (group 1), 0.6 (group 2), and 0.9 mg/kg (group 3), administered IM for periods of 15, 30, and 45 days, respectively. In this study, animals that received EB at a dose of 0.3 mg/kg for 15 days were the most successful in induction, presenting significant changes (*p* < 0.01) in both uterine coefficient and smooth muscle thickness compared to the control group.

The administration of estrogen associated with progestogens appears to be more effective in the development of hormone-induced UL when compared with estrogen alone and has been used in different protocols adapted to their experimental needs^9,11,13–16,18,20–26^.

Li et al.^13^ used Sprague-Dawley rats and administered estradiol benzoate (0.5 mg/kg) IM on Mondays, Wednesdays, and Fridays for 12 weeks, and at week 13, introduced progesterone (5 mg/kg) diluted with estradiol benzoate, maintaining this regimen until week 15. Similarly, Yamei et al.^18^ administered intramuscular progesterone (1 mg/kg) on Mondays, Wednesdays, and Fridays, complemented with EB (2 mg/kg) applied on Tuesdays, Thursdays, and Saturdays for eight weeks.

Adding this approach, Yu et al.^15^ employed diethylstilbestrol (0.167 mg/kg) by gavage daily and progesterone (1 mg/kg) IM once a week for 20 weeks, indicating that the route of administration can vary depending on the objective of the experiment. Similarly, Yuan et al.^24^ applied estradiol benzoate (0.5 mg/kg) intraperitoneally daily and progesterone (4 mg/kg) weekly for five weeks, followed by an additional one-week period with both hormones at the same dose and route of administration. Zeng et al.^25^ administered estradiol benzoate (0.5 mg/kg) intramuscularly three times a week for eight weeks and, subsequently, introduced progesterone (4 mg/kg) intraperitoneally twice a week for another four weeks, demonstrating a phased induction model.

Zhang et al.^20^ applied diethylstilbestrol (1.35 mg/kg) by gavage daily and progesterone (1 mg) intramuscularly for five weeks. Gao et al.^26^ administered estradiol benzoate (0.5 mg/kg) IM, combined with progesterone (1 mg/kg) daily for nine weeks, highlighting a simple and continuous protocol. Chen et al.^21^ followed a split regimen, using estradiol benzoate (0.5 mL/kg) IM for eight weeks, followed by the introduction of progesterone (0.25 mL/kg) intraperitoneally, twice a week, for four weeks. The study by Lin et al.^14^, which used Institute of Cancer Research (ICR) mice (CD-1), started with diethylstilbestrol (0.4 mg/kg) by daily gavage for four weeks, and then this compound was associated with medroxyprogesterone 17-acetate (5 mg/kg) by the same route for another four weeks.

Lin et al.^16^ followed a simple and continuous protocol, using daily injections of estradiol benzoate (0.2 mg) and progesterone (0.2 mg) for 60 days in specific pathogen-free (SPF) mice. Qiu et al.^22^ applied estradiol benzoate (0.5 mg/kg) daily intraperitoneally and progesterone (4 mg/kg) weekly for 10 weeks in Sprague Dawley rats, adding both hormones simultaneously in the last five days. Still dealing with hormonal induction, Shi et al.^23^ administered estradiol benzoate (0.5 mg/kg) IM for 35 days and progesterone (1 mg) daily, managing to induce the model in only 14 days, evidencing the efficiency of their approach.

Finally, Liu et al.^11^ chose to use C57B6 mice, which received intramuscular injections of estradiol benzoate at 0.05 mg/100 g three times a week for 12 weeks. After this period, the animals began receiving intramuscular progesterone injections twice weekly for another four weeks.

The main advantage of hormonal methods is their simplicity compared to surgical or genetic approaches. Administration of estrogen alone or in combination with progestogen allows precise control of doses and exposure time, in addition to not requiring invasive procedures. This factor reduces stress and the risk of direct complications and is less aggressive. The costs associated with hormones are relatively low, and the technique requires only basic training from the team in administering substances orally, IM, or intraperitoneally.

The limitations of this model are due to the prolonged use of high doses of hormones that can cause undesirable systemic effects, such as metabolic, hepatic, or renal changes; the development of UL may vary between individuals, requiring a larger number of animals to ensure an adequate sample; the lack of confirmation before starting the experimental research, in most of the studies evaluated; this model may not fully reflect the etiological complexity of UL in humans, which also involve genetic, inflammatory and environmental factors; and finally, there is no standardization regarding the type, associations, doses, route of administration, and time of use of the hormones used.

### Monosodium glutamate

MSG, a non-essential amino acid commonly used as a flavor enhancer in processed foods, was used alone to induce UL by increasing cholesterol and consequently elevating estrogen and progesterone levels, leading to uterine hyperplasia and development of UL, with daily doses of 200 mg/kg administered orally for 30 days in Wistar or Sprague Dawley rats^28–31^.

Mamoon et al.^32^ used a different scheme in Wistar rats, administering MSG 200 mg/kg in the first week, 400 mg/kg in the second week, and 600 mg/kg from the third week until the eighth week when the experiment ended. In addition, estradiol benzoate (200 µg) was injected subcutaneously twice a week from the third week until the end of the experiment.

The models’ induction took two to eight weeks, depending on each study’s protocol. A significant advantage of using MSG for UL induction in rats is the relatively low cost and easy availability of the compound, making the model accessible to laboratories with limited resources. In addition, MSG is administered orally, a less invasive and more practical method, which can reduce animal stress compared to surgical interventions or more complex techniques. The relatively short time for UL induction, ranging from two to eight weeks, is another important advantage, allowing rapid results^28–32^.

On the other hand, the mechanism of action of MSG in inducing UL by hormonal increase is not entirely elucidated, which may limit the translation of findings to human models. Thus, administering daily doses for prolonged periods requires intensive management, which may increase variability between experimental groups due to factors such as stress or absorption difficulties.

### Ischemic injury

Another model of UL development is an ischemic injury, because it induces tissue fibrosis, in which uterine scarring and remodeling occur through the characteristic of myofibroblast transdifferentiation and scar formation^33^.

It has been reported that mast cells could promote fibrosis, which is intrinsically linked to the pleiotropic cytokine transforming growth factor (TGF)-β, that plays an important physiological role in the initiation and control of fibrosis, in addition to being a key mediator of fibroblast activation and driving an abnormal extracellular matrix synthesis in fibrotic diseases^9,34^.

The ischemic injury technique is based on surgically exposing the rat’s abdomen and clamping the celiac artery for 30 minutes^33^. This method has the advantage of being able to study the effects of hypoxia and vascular injury on the pathogenesis of UL, providing valuable information on the contribution of the ischemic environment to tumor development. This model could assist in investigating molecular mechanisms and evaluating therapeutic interventions to address changes in the uterine microenvironment caused by ischemia.

The limitations of ischemic injury include its highly invasive nature, which significantly compromises animal welfare, increasing the risk of pain, stress, and postoperative complications. In addition, ischemia can affect other organs and vascular systems, introducing confusion in the results and making it difficult to attribute the effects exclusively to the uterus. Despite being a method of inducing UL, it appears to be the furthest from preserving animal welfare, causing injury, requiring extensive surgical preparation, and possibly affecting the animal’s vascular system^33^.

### External stimulus

The studies conducted by Zhao et al.^35^ and Feng et al.^36^ demonstrated that external stimulation effectively enhances UL induction in rats subjected to hormonal treatments with estrogen and progesterone. Their findings indicate that providing daily external stimuli for approximately two weeks significantly improves UL development in these animals.

In traditional Chinese medicine, UL treatments promote blood circulation and remove uterine stagnation. This stagnation is due to disturbing emotions such as anger and long-term concern, justifying the induction of UL through external change and environmental factors such as light, noise, and temperature to enrage rats by mimicking the depressed and angry states of humans^35^.

Sprague Dawley rats received 0.9 mg/kg/day of epinephrine hydrochloride IM from the fourth week onwards and received external stimulation daily four hours after injection. The stimuli were exposure to 60 dB of noise for 3 hours, reversal of the day and night cycle in one day, swimming in water at 5–10°C for 4 minutes, being hung by the tail for 10 minutes, and being exposed to the heat of 50°C for 10 minutes, with each stimulus being performed at least twice for a two-week cycle^35,36^. Although these studies convey an idea of greater efficiency with these methods, these practices dangerously resemble animal abuse.

### Cellular autotransplant

The transplantation of ELT-3 cells, a UL cell line derived from Eker rats, was used in five studies involving mice^37–42^.

Abdelaziz et al.^37^ and Halder et al.^40^ used sustained-release hormone pellets containing 17β-estradiol (90 days), implanted four days before cell transplantation in athymic nude mice and nude mice (Foxn1nu), respectively. Bar-Joseph et al.^38^ also combined ELT-3 cell implantation with hormonal induction in nude athymic mice by administering beta-estradiol 17-valerate (2 μg) two days before the cell transplantation procedure. Lin et al.^14^, Chen et al.^39^, and Lin and Wu^41^ performed only the cell implantation without hormonal supplementation in nude mice (Foxn1nu).

This method is efficient for inducing UL in mice and is comparable to the hormonal method alone in terms of results. In addition, cell transplantation is less invasive and less stressful since it involves only the injection of cells, dispensing with more complex procedures. In addition, the procedure is faster, reducing animal stress, because it involves only the injection of cells. The method also offers flexibility and can be used with or without hormonal supplementation, depending on the needs of the study^14,37–41^.

However, dependence on the ELT-3 cell line requires specific laboratory conditions for its maintenance, which can be a logistical challenge. The model is also largely validated in mice, which limits its application in other species. Furthermore, because the cells are derived from rats, there are limitations in directly translating the findings to humans.

Another cell autotransplant model is the VX2 tumor in rabbits, which uses the VX2 tumor line, an anaplastic squamous cell carcinoma derived initially from papillomas induced by the Shope papillomavirus, and can be transplanted from one animal to another and into any tissue, including the myometrium of rabbits^43^.

Rhee et al.^44^ evaluated the feasibility of this model in the study of the treatment of UL in rabbits through uterine artery embolization. To create the model, the authors performed a laparotomy to expose the uterus and injected 0.3 to 0.5 mL of a solution containing VX2 cells, obtained from tumors of donor rabbits, into the uterine horn of six rabbits.

After model confirmation, magnetic resonance imaging (MRI) scans with and without contrast were performed before and after uterine arterial embolization (UAE). The gadolinium-based contrast was administered manually, followed by saline, and images were captured 2 minutes after injection. After the MRI, the rabbits were taken to the angiography suite for the UAE procedure, guided by fluoroscopy^44^.

Among the characteristics of this model, it does not require immunocompromised or transgenic animals. Therefore, it can be used in healthy animals and allows tumors to be obtained in a few weeks with > 95% efficiency. Another decisive factor for the use of this model in rabbits is the larger size compared to rodents, allowing the use of medical devices similar to those used in humans, which allows the evaluation of locoregional therapies, including transarterial chemoembolization, radioembolization, thermal ablative therapies, and combined approaches^43^.

Nevertheless, it is a valuable model for studying the interaction between tumor microenvironment and neovascularization and may assist in the development of therapeutic strategies aimed at reducing blood supply to the UL^45^.

### Xenotransplantation

Xenotransplantation is the transplantation of organs, tissues, and cells between organisms of different species^46^. Based on this knowledge, different studies^47–54^ have investigated human UL xenotransplantation in rats and mice as an experimental model for the study of UL. This approach requires the administration of high doses of hormones and, in many cases, immunosuppression of the animals to avoid transplant rejection.

In the study conducted by Sousa et al.^53^, Wistar rats were immunosuppressed with mycophenolate mofetil (40 mg/kg) administered by oral gavage, starting 15 days before xenotransplantation and maintained until the end of the experiment. After immunosuppression, the animals underwent surgery to transplant UL tissue fixed on the right side of the peritoneal cavity or in the subcutaneous tissue of the right flank. After the procedure, estradiol valerate (1 mg) was administered as a hormonal supplement, dissolved in 250 mL of water, and offered *ad libitum*, with daily replacement. This study reports a new, practical, and relatively inexpensive model of UL in rats, but such a model could be improved by studying the effects of progesterone supplementation and more accurately assessing the mode of estrogen administration^45^.

Another technique includes the implantation of a pellet containing 17β-estradiol (0.05 mg) and progesterone (50 mg) into the subcutaneous tissue during surgery^54^ or 15 days before transplantation^47,52^. Alternatively, Corachán et al.^48^ performed the xenograft of two human UL fragments in the peritoneal cavity (one on each side) associated with ovariectomy and subcutaneous implantation of pellets containing 17β-estradiol (0.36 mg) in the neck of the animals, complementing the protocol with the administration of progesterone (1 mg/day) for one week, followed by a two-week interval to simulate the menstrual cycle, maintaining this regimen for 60 days.

The study by Huang et al.^49^ investigated the role of estrogen and laparoscopic surgery on the growth of parasitic fibroids, an extrauterine leiomyoma, in severe combined immunodeficiency (SCID) mice by xenotransplantation of 10 fibroids fragments (1–2 mm) distributed across the four quadrants of the peritoneal cavity. Three primary induction methods were used: xenotransplantation without ovariectomy (group 1), xenotransplantation with bilateral ovariectomy (group 2), and xenotransplantation with estradiol (E2) supplementation (group 3). In the first group, estradiol-supplemented fibroid fragments were cultured and transplanted into the animals. In addition, the animals underwent a CO_2_ insufflation procedure at 4 mmHg for 10 minutes to simulate laparoscopic conditions. The second group underwent bilateral ovariectomy two weeks before xenotransplantation, serving as a model of estrogen deprivation to evaluate the influence of the hormone on fibroid implantation. The third group received exogenous estradiol supplementation after xenotransplantation to verify whether the increase in hormone levels favored the implantation and growth of fibroids. The mice in group 3 presented the highest number of implants and a total weight of implanted fibroids, in addition to a significant increase in the expression of markers of cell proliferation, angiogenesis, and hormone receptors. In contrast, the mice submitted to ovariectomy had fewer implantations and lower growth of fibroids, indicating that estrogen depletion significantly reduces the proliferation and angiogenesis of implanted fibroids.

Lee et al.^50^ implanted human UL cells mixed in Matrigel and prolonged-release hormone pellets with 17b-estradiol (0.05 mg/90 days) and progesterone (25 mg/60 days) into the abdominal subcutaneous tissue of mice, concluding the experimental model in eight weeks when they started treatment with flavopiridol. Zakaria et al.^55^ implanted human leiomyosarcoma cells (20 μL containing 106 cells) into the subcutaneous tissue of the right flank of nude mice. The experiment ended 21 days after transplantation.

Koohestani et al.^56^ and Qiang et al.^57^ performed the implantation of slow-release hormone pellets in subcutaneous tissue with estradiol and progesterone (E2 0.8 mg + P4 75.2 mg) + cholesterol (4 to 5 mg) associated with ovariectomy and implantation of human UL in the renal capsule of NOD-scid IL2Ry^null^, establishing the model after four weeks.

In addition to these approaches, Fritsch et al.^58^ performed xenotransplantation of human UL tissue into the subcutaneous tissue of the ventral region of immunodeficient SCID mice. The mice were ovariectomized and, during tissue transplantation surgery, received subcutaneous implants of estradiol (0.05 mg/90 days) and progesterone (25 mg/60 days) hormone pellets to simulate the human hormonal environment. Graft growth was assessed between 15 and 60 days after transplantation, and greater proliferation was observed in the presence of E2 and P4 combined when compared with the results of the groups without hormone supplementation or with E2 supplementation alone.

Li et al.^51^ administered bisphenol A (BPA) orally at the dose of 400 μg/kg body weight per day, starting two weeks before cell injection and continuing until the 42nd day after subcutaneous injection of human UL (1×10^7^ cells) into the right dorsal flank.

This approach, which uses transplantation and hormonal supplementation without immunosuppression, was mainly used in mice of the BALB/C nude, NOD-SCID, or NOG (NOD/Shi-scid/IL2Rγ^null^) lineages, which present natural immunosuppression, eliminating the need for immunosuppressive drugs and reducing the chances of tumor rejection.

These methods are particularly valid for the experimental induction of UL, as they use human tissue, allowing greater translational relevance when evaluating drugs intended for human patients. In addition, it is possible to reproduce biological characteristics and therapeutic responses similar to those observed in humans, offering realistic modeling.

Whether through pellets or enriched water, hormone supplementation allows efficient control of the hormonal environment necessary for tumor growth. In immunosuppressed strains, such as BALB/C nude or NOG, the need for immunosuppressive drugs is reduced, simplifying experimental management. However, the technique is invasive and stressful for the animals, involving surgical procedures, anesthesia, analgesia, and postoperative care.

### Spontaneous

The study by Laik-Schandelmaier et al.^59^ analyzed 83 female guinea pigs that underwent histopathological examinations, of which 64 had surgically removed uterine masses and 19 underwent complete necropsy. Among the cases evaluated, 18 leiomyomas (21.7%) and nine leiomyosarcomas (10.8%) were diagnosed, demonstrating a predominance of benign tumors over malignant ones. In addition, 20 endometrial adenomas (24.1%), three adenocarcinomas (3.6%), and other alterations such as cystic glandular hyperplasia and cervical polyps were observed. These findings suggest that guinea pigs can spontaneously develop UL and other reproductive tract tumors, which could make them potential experimental models for studying the disease.

However, the lack of information on the hormonal regulation of tumors in these animals poses challenges to their use in controlled research. The need to standardize factors such as age, hormonal cycle, and response to treatment should also be considered before validating the model^59^.

In addition to conventional models, other methods that do not fall within the definition of induced models have been explored due to their ability to develop UL spontaneously and may contribute significantly to translational research in the future. Among these alternative models, studies with potbellied pigs^60^, aged hens^61,62^, chimpanzees^63,64^, rhesus monkeys^65^, and Guianan squirrel monkeys^66^ stand out, each with its particularities in the induction and development of UL.

Another alternative is the potbellied pig (*Sus scrofa*), which has been studied due to the similarity of the porcine uterus to the human uterus concerning macroscopy, cellular morphology, level of mitotic activity, collagen deposition pattern, hormonal response, and incidence similar to that found in women. An additional point is the duration of the swine estrous cycle, which occurs every 21 days and lasts approximately two or three days^45^.

A study by Mozzachio et al.^60^ evaluated 106 female potbellied pigs using medical records provided by a local potbellied pig spay/neuter program, pig sanctuaries, and the Duchess Fund database for evidence of reproductive disease or surgery for spontaneously occurring fibroid-like tumors in potbellied pigs. Among the animals analyzed, 17 cases of spontaneously developing neoplasms were identified, but only 13 received histological evaluation, which identified 11 UL, one leiomyosarcoma, and one undifferentiated sarcoma. The authors reported difficulties, such as the lack of histological analysis for all detected neoplasms, standardization of tissue sampling and fixation methods, macroscopic description of lesions, and clinical signs.

Regarding hormone receptors, it was found that healthy tissues and UL from potbellied pigs presented, in decreasing order of immunoexpression, progesterone receptors (PR), estrogen receptor-α (ER-α), and estrogen receptor-β (ER-β), a pattern that is also observed in women. Although the relationship between the expression of sexual steroid hormones and UL is not entirely clear yet, most studies indicate that there is an increase in the immunoexpression of these tumors in affected tissues, a fact that has not been proven yet in potbellied pigs^60^. Thus, this species has great potential for developing an animal model of UL, but new studies are necessary to evaluate the feasibility of this method.

The aging hen (*Gallus gallus domesticus*) model has been explored as an alternative for the study of UL since up to 60% of these birds develop UL of the ventral ligament of the oviduct spontaneously, varying according to the lineage and breed^45^.

Aiming to evaluate the possibility of using this species as a model of UL, Machado et al.^62^ evaluated 263 hens without signs of disease. The research revealed a higher prevalence of UL, in addition to an increase in the size and quantity of tumors, especially in the third and fourth years of laying. These tumors present similarities with human ULs, especially when it comes to the expression of the Bcl-2 protein, the presence of smooth muscle actin and desmin in immunohistochemistry, the relation ER/PR, and localized cell proliferation^45,61^.

Thus, the data suggest that the aged chicken is a potential model to study the pathophysiology of ULs and evaluate new therapies. The efficacy of this model could be improved with noninvasive detection methods and continuous monitoring without sacrificing the animal, but further studies are needed to assess this feasibility^45^.

The spontaneous development of UL in chimpanzees has been reported in the literature, with studies addressing their prevalence, histopathology, and possible hormonal influences. In a study conducted in two primate facilities in the United States of America, it was observed that, of the 195 females analyzed with ages ranging from 15 to 52.5 years old (mean = 28.47), 28.2% (n = 55) had UL, with a mean age of 30.4 at the time of diagnosis. The prevalence increased to approximately 40% in females over 30 years old, suggesting a relation between advanced age and tumor development^64^.

The diagnosis was made primarily by physical examination (palpation) and transabdominal or transrectal ultrasonography during annual examinations under sedation. Histopathology confirmed the presence of interlaced bundles of smooth muscle, with some areas of necrosis, vascular embolization, and inflammatory cell infiltration. The influence of sex hormones on the formation of these tumors has also been investigated. The use of progesterone-based hormonal contraceptives was associated with a significant reduction in the prevalence of UL, suggesting a possible protective effect^64^.

Another study reported a case of UL in a 22-year-old female chimpanzee who presented with anorexia, frequent vomiting, and dehydration. During necropsy, an enlarged uterus was observed, with the lumen obstructed by firm, whitish nodules that extended through the myometrium. Immunohistochemical analysis revealed strong expression of estrogen and PR, suggesting a hormonal relationship in the tumor development. These findings indicate that the chimpanzee may be a relevant model for the study of UL, given that the tumors share histological and hormonal characteristics with those found in humans. However, further studies are needed to understand better the underlying mechanisms and the response to different therapies^63^.

The rhesus macaque (*Macaca mulatta*) has been identified as a relevant model for the study of spontaneous neoplasms, including UL. These animals share several physiological similarities with humans, including the relationship between aging and increased incidence of tumors. The analysis conducted in two colonies of rhesus macaques (n = 217) kept in captivity revealed that UL represented the most common urogenital neoplasm, corresponding to 30 of the 39 cases identified in the uterus of these females. The mean age at diagnosis was 25.1 years old, ranging between 15.0 and 34.8, suggesting a correlation between advanced age and the development of these lesions^65^.

UL in rhesus monkeys is described as a benign smooth muscle tumor, similar to those found in women, which reinforces their usefulness as an experimental model for investigations into pathophysiology and possible therapeutic approaches. Furthermore, cases of well-circumscribed neurilemmomas within UL have been reported, an unusual finding that may have implications for understanding tumor biology in these primates^65^.

The high incidence of UL in these primates and their similarity to human tumors make the rhesus monkey a promising model for research on the pathogenesis and treatment of UL, although additional studies are needed to clarify the factors involved in the development of these tumors. The first documented case of UL in Guiana squirrel monkeys (*Saimiri sciureus*) was described in a 12-year-old adult female who presented with a firm, palpable mass in the caudal region of the abdomen^66^.

Due to the size and rapid growth of the lesion, an exploratory laparotomy was performed, revealing an intraluminal tumor in the uterus, which was removed by partial hysterectomy. Macroscopically, the tumor was firm, non-encapsulated, reddish pink in color, and had a smooth surface. Histological analysis confirmed a UL composed of intertwined bundles of smooth muscle cells with elongated, cigar-shaped nuclei and low mitotic activity^66^. This report reinforces that, although rare, UL can occur in Guiana squirrel monkeys, a species that should have potential as a model of experimental UL. However, as with other spontaneous models, further studies are needed to assess the similarity of these tumors between primates and humans.

### Induction proof

An essential consideration in developing an effective experimental method for UL induction is the implementation of a reliable verification process. This is crucial, as any of the above-mentioned methods may fail, compromising the accuracy and reliability of the results obtained. The review found that 34 studies did not clarify how UL development was confirmed.

Less than half of the induced or spontaneous ULs models (n = 22) confirmed the presence of induction before the start of experimental drug use^19,21,23–25,32,37–41,44,47,48,50,51,53,54,56,59,64,66^. Halder et al.^40^ and Lee et al.^50^ verified it from a macroscopic point of view, Abdelaziz et al.^37^, Chen et al.^39^, Bar-Joseph et al.^38^, Lin and Wu^41^, Borahay et al.^47^, Corachán et al.^48^, Lee et al.^50^, Li et al.^51^, and Suzuki et al.^54^ measured the induction with a caliper, externally of the animal. Meanwhile, other authors chose to use ultrasound in isolation^47,64,66^ or Doppler flowmetry^32,44^, which showed higher blood flow velocities and lower Doppler indices in rats that developed UL. This approach is manageable in rodents and primates, as UL is visible as an abdominal, axillary, or dorsal bulge, depending on the tumor’s location, and is easily detected on imaging.

In a complementary manner, Long et al.^66^ performed radiography, while Corachán et al.^48^ followed another approach to demonstrate UL employing positron emission tomography with computed tomography (PET/CT), using the radiopharmaceutical 2-deoxy-2-[18F]fluoro-D-glucose (18F-FDG) on days 21 and 60 of the experiment, to evaluate the therapeutic effects of vitamin D in the short and long term, respectively. This technique allowed the detection of UL xenografts from glucose uptake by tumor cells.

Six other studies performed surgeries to evaluate UL induction with macroscopic visualization in tissues in all animals^19,21,24,25,53,66^. Yousefi et al.^19^, Chen et al.^21^, Yuan et al.^24^, Sousa et al.^53^, Koohestani et al.^56^ and Long et al.^66^ evaluated two or three animals from each group. Zeng et al.^25^ confirmed euthanasia induction and performed a histopathological analysis of the model group, which was considered successful if the smooth muscle layer of the uterus showed significant thickening and fibrosis.

Laik-Schandelmaier et al.^59^ evaluated the presence of UL through histopathological analysis of tumors removed during surgery. A single study performed MRI to evaluate the success of tumor induction in rabbits^44^.

Although induced animal models are widely used in UL studies, they have significant limitations, such as physiological and hormonal differences compared to humans and variations in induction methods and response to stimuli. On the other hand, spontaneous models, such as those reported in primates and other animals, may offer an alternative closer to the disease’s natural pathogenesis. However, the scarcity of detailed studies on these cases and the difficulty of standardization limit their immediate application as experimental models. Future investigation of these models could provide a more translational and complementary approach to induced models, increasing the relevance of the findings for clinical practice.

### Ethical considerations

The analysis of experimental models revealed a dichotomy between the need to mimic human pathophysiology and the ethical considerations of animal welfare. The validation of any experimental model must necessarily align with the principles of the 3Rs (Replacement, Reduction, and Refinement), as classically established by Russell and Burch^67^ and standardized by the Brazilian Guideline for the Care and Use of Animals (CARE)^68,69^. From this perspective, induction methods that employ swimming in ice water, tail suspension, exposure to extreme noise, and other animal-stress techniques require rigorous ethical evaluation. The CARE classifies procedures involving immobilization stress or forced swimming with physical exhaustion as having a severe degree of invasiveness (G3 or G4), since they cause severe pain, suffering or stress and hinder the expression of natural behaviors^69^.

Such practices resemble mistreatment and violate the principle of refinement, which requires that proposals be designed to avoid pain and stress, and that the absence of less invasive alternatives be thoroughly proven^69^.

Furthermore, models based on ischemic injury and prolonged supraphysiological hormonal administration present significant ethical limitations. Surgical ischemia poses risks of postoperative pain and systemic compromise, requiring intensive monitoring and rigorous analgesia to reduce suffering, as recommended by postoperative care guidelines. Regarding reduction, it is observed that many protocols still depend on the euthanasia of large groups of animals for tumor evaluation^69^. The implementation of noninvasive imaging technologies (such as ultrasound and MRI) would allow longitudinal follow-up of the same individual, significantly reducing the number of animals used without compromising the statistical reliability of the results. Therefore, the advancement of translational research in leiomyomas should prioritize spontaneous or xenotransplantation models that, combined with noninvasive monitoring methods, respect the ethical imperative of minimizing harm and maximizing well-being, and reject induction protocols that inflict unjustifiable severe suffering.

## Conclusion

This scoping review synthesized evidence on animal models of UL, mapping induction methods (transgenic, hormonal, MSG, ischemic injury, external stimulus, cell transplantation with the ELT-3 and VX2 tumor lineage, and xenotransplantations in tissue and cellular forms), species used (rats, mice, potbellied pigs, hens, chimpanzees, rhesus monkeys, and squirrel monkeys), and evaluation strategies (macroscopy, microscopy, PET/CT, radiography, MRI and ultrasonography with and without Doppler flowmetry). Thus, substantial heterogeneity was observed regarding the species used, induction protocols, hormonal regimens, confirmation methods, and outcomes evaluated, which significantly limits reproducibility and hinders direct comparison between studies.

To provide practical guidance based on this synthesis, we established that the choice of model should be guided by the specific objective of the investigation. Mechanistic studies in genetics and molecular biology should prioritize transgenic models associated with hormonal induction (such as Eker rats), as they mimic the interaction between genetic mutations and endocrine stimuli, approximating human etiopathogenesis. In addition, basic pharmacological investigations and initial screening find in rodent hormonal models a low-cost solution, simplicity, and precise dose control, although pharmacokinetic studies requiring the use of medical devices or hemodynamic monitoring are more appropriately performed in larger animals, such as the VX2 tumor in rabbits or porcine models. Finally, for preclinical efficacy trials of new drugs, the current gold standard is xenotransplantation models with human tissue or cells, which offer the greatest translational relevance by replicating the tissue architecture and the actual therapeutic response observed in women.

Among the models identified, hormonal models in rodents stand out for their accessibility, low cost, and applicability in mechanistic and pharmacological investigations. In contrast, xenotransplantation models and spontaneous models, especially in pigs and non-human primates, demonstrate greater translational relevance due to their greater anatomical, histological, and hormonal similarity to human leiomyomas. Therefore, future research should prioritize the establishment of standardized, ethically responsible, and reproducible protocols for the induction and confirmation of leiomyomas in animal models, preferably incorporating noninvasive imaging methods, such as ultrasound and MRI, combined with histopathological validation. Furthermore, increased investment in research on spontaneous models could provide valuable information on the natural history and pathophysiology of fibroids, contributing to the development of more effective and less invasive therapeutic strategies for women affected by this condition. Standardization of experimental models and outcome measures is fundamental to increasing the translational impact of this research and accelerating the development of medical therapies capable of reducing the clinical, social, and economic burden of UL worldwide.

## Data Availability

All relevant data and details of resources can be found within the article.
